# Exposure-Aware Training for Low-Light Object Detection Without Target-Domain Data

**DOI:** 10.3390/jimaging12060245

**Published:** 2026-05-29

**Authors:** Yawen Su, Min Lu

**Affiliations:** School of Intelligence Science and Technology, Inner Mongolia University of Technology, Hohhot 010080, China; 202310201033@imut.edu.cn

**Keywords:** low-light object detection, domain generalization, exposure-aware training, synthetic degradation, zero-inference adaptation

## Abstract

Low-light object detection remains challenging because insufficient illumination obscures visual features and increases the discrepancy between training and testing conditions. Existing approaches often rely on detector redesign, image enhancement, or target-domain data, which may introduce additional complexity during training or inference. This paper presents Exposure-Aware Training (EAT), a lightweight degradation-based training strategy that applies illumination attenuation and additive Gaussian noise to normal-light images during training. The degradation parameters are estimated from real low-light image pairs, while the detector architecture remains unchanged. Our experimental results show that moderate degradation consistently improves low-light detection performance, whereas excessively strong degradation may damage semantic information, especially for small objects. Under both cross-domain and mixed-training settings, EAT achieves stable improvements on YOLOv8 and Faster R-CNN, with more noticeable gains for illumination-sensitive categories. These results indicate that exposing detectors to task-oriented illumination degradation during training can effectively improve low-light detection performance without additional inference overhead.

## 1. Introduction

Low-light object detection is important for applications such as nighttime surveillance, autonomous driving, and intelligent transportation systems. Compared with normal-light conditions, detection performance under low illumination usually drops noticeably because insufficient lighting reduces image contrast, weakens texture details, and introduces noise, making feature extraction less stable. In practice, the collection and annotation of real low-light datasets are also expensive, which further limits the applicability of fully supervised approaches.

Current low-light detection approaches mainly fall into three categories. One line of work focuses on improving image visibility before detection using enhancement models such as EnlightenGAN [1] and Zero-DCE [2]. Although these methods often improve perceptual image quality, their optimization objectives are usually designed for visual appearance rather than detection robustness, and the enhancement process may alter the original feature distribution. Another direction focuses on improving low-light performance through detector redesign, e.g., by introducing attention modules, feature enhancement blocks, or multi-branch architectures. Such methods can improve detection accuracy but often increase architectural complexity and inference cost. A third direction focuses on adopting domain adaptation or domain generalization strategies to reduce the distribution gap between normal-light and low-light domains. These methods typically rely on target-domain data or additional alignment constraints during training.

This work studies low-light detection from a degradation-oriented training perspective. Instead of restoring visually clearer low-light images, we focus on whether controlled illumination degradation during training can improve robustness to illumination variation. For detection tasks, exposure to different illumination conditions during training may be more important than recovering perceptually pleasing images. From the viewpoint of object detection, preserving task-relevant structures may be more important than accurately reproducing visually realistic low-light images.

Based on this idea, we propose Exposure-Aware Training (EAT), a training-stage degradation strategy for low-light object detection. EAT constructs degraded samples by applying illumination attenuation and noise perturbation to normal-light images during training, encouraging the detector to learn more illumination-stable representations. Different from conventional random photometric augmentation, the degradation parameters are estimated from statistics of real low-light image pairs rather than selected entirely heuristically. This method can be integrated into existing training pipelines without modifying the detector architecture or introducing additional inference-stage computations.

Experiments across multiple low-light settings show that EAT produces stable but generally moderate improvements. Under the strict VOC→ExDark zero target-domain setting, the method achieves consistent cross-domain gains without using any ExDark images during training. When limited real low-light data are available, mixing EAT-generated degraded samples with real images further improves performance. Additional experiments on category-level behavior, detector transferability, and nonlinear illumination perturbation suggest that the method improves robustness mainly by increasing exposure diversity during training.

The main contributions of this work are summarized as follows:

1. A lightweight degradation-based training strategy, termed Exposure-Aware Training (EAT), is introduced to improve low-light robustness without modifying detector architectures.
2. The influence of degradation intensity on detection performance is systematically analyzed, showing that moderate degradation improves robustness, while overly strong degradation damages semantic information.
3. We validate the method under cross-domain, mixed-training, and multi-detector settings and further compare it with conventional photometric augmentation strategies.

## 2. Related Work

This section reviews relevant studies from three perspectives: low-light object detection, low-light image enhancement, and synthetic-data-based domain generalization. We analyze the limitations of existing approaches to position our method.

### 2.1. Low-Light Object Detection

ExDark [3] is a real low-light dataset covering diverse scenes, and it is commonly used for evaluation in this task. Datasets such as DARK FACE [4] are designed for specific scenarios (e.g., face detection). Early methods often used a two-stage pipeline: applying image enhancement first and then feeding the output to a standard detector, effectively treating low-light detection as a preprocessing step.

In recent years, researchers have attempted to improve models’ adaptability to low-light environments through architectural design. For instance, Hashmi et al. proposed FeatEnHancer [5] to enhance hierarchical features for low-light object detection; Peng et al. [6] introduced a fusion feature enhancement method based on YOLOv5; Hong et al. proposed YOLA [7] to learn more illumination-robust representations; Ci [8] developed a multi-module enhanced detection framework combining image enhancement and adaptive feature fusion; and the creators of 3L-YOLO [9] implemented improvements based on YOLOv8n using switchable atrous convolution (SAConv) and a dynamic detection head.

Although these architectural modifications provide certain improvements, they usually rely on low-light annotated data during training and often introduce additional inference costs or model complexity. The current strategy does not depend on architectural modification or target-domain data; instead, it helps the model to learn illumination-robust features through controllable degradation during training. In addition, when a small amount of real low-light data is available, EAT-degraded images can serve as a data augmentation strategy with which to further improve performance. However, most existing architecture modification methods rely on low-light annotated data and increase model complexity, which may limit their applicability in data-scarce or resource-constrained scenarios.

### 2.2. Low-Light Image Enhancement and Its Application in Detection

Low-light image enhancement methods are designed to improve the visual quality of images. Classic approaches such as Retinex [10] adjust brightness by decomposing illumination and reflectance components, while subsequently designed methods like LIME [11], Zero-DCE [2], and EnlightenGAN [1] achieve enhancement through learning-based approaches.

Although these methods have achieved success in visual perception, their gains for object detection are inconsistent. Enhancement is optimized for human perception rather than preserving task-related features. As a result, enhancement may distort important cues such as edges, textures, and small objects that are critical for detection. In addition, some methods introduce artifacts that further reduce detection performance. Moreover, enhancement is usually applied during testing as a post-processing operation. Although this may improve image appearance, it does not address the fundamental mismatch between training and testing distributions.

Here, the detector operates entirely during training by introducing controlled degradations, achieving feature-level adaptation to illumination changes without test-time overhead. Most enhancement methods are designed primarily for visual perception rather than detection-oriented feature preservation, and their benefits for detection under different low-light conditions may be inconsistent [12,13]. In addition, test-time enhancement cannot reduce the domain gap between training and testing distributions.

### 2.3. Domain Generalization and Synthetic Data

Domain generalization is intended to maintain model performance under unseen conditions [14,15]. Existing approaches mainly fall into three categories: general data augmentation, synthetic data generation, and feature invariance learning. General data augmentation. Methods such as AugMix [16] and photometric distortion improve robustness by introducing random perturbations, including changes in brightness, contrast, and color. However, these transformations are largely task-agnostic, and they are not explicitly designed to address the factors that degrade detection performance in low-light conditions. As a result, while they increase data diversity, they may not effectively resolve illumination-specific challenges. Unlike generic augmentation methods that mainly increase perturbation diversity, EAT focuses specifically on illumination-related factors that directly affect detection performance.

Unsupervised domain adaptation for low-light detection. Xiong et al. [17] proposed fine-tuning feature interaction for unsupervised domain adaptive low-light detection. This approach reduces annotation requirements but still requires unlabelled target-domain images during training. Zero-shot domain adaptation methods [18] further eliminate the need for target-domain data, but they often suffer from larger performance drops under extreme illumination variations. Synthetic data generation. To alleviate the scarcity of annotated low-light data, some methods use generative models to produce synthetic images. For example, CycleGAN-based methods perform day-to-night style transfer, while simulation platforms such as CARLA generate synthetic scenes under different illumination conditions. Although these methods can produce large-scale training data, they often rely on complex models and may still suffer from the distribution gap between synthetic and real data. In addition, their objective is usually visual realism rather than task relevance. For images generated by EAT, task-oriented degradation is prioritized rather than visual realism. One drawback of synthetic data generation methods is that they usually require complex generative models and may still suffer from the gap between synthetic and real domains, whereas EAT adopts a simple task-oriented degradation that directly targets detection-related factors.

In this study, we adopt a task-oriented perspective. Instead of introducing arbitrary or visually driven transformations, we only model the key degradation factors that directly affect detection performance: illumination attenuation and additive noise. These factors are estimated from paired data, reducing reliance on heuristic parameter selection and avoiding the use of complex generative models.

### 2.4. Method Positioning and Distinctions

In this paper, we define low-light object detection as a task-oriented degradation-modeling problem, where the choice of transformation is guided by its impact on detection performance rather than visual quality or data diversity. Table 1 compares the key characteristics of different low-light detection approaches.

EAT differs from previous methods in several important aspects: it operates entirely during training without introducing inference-stage overhead, avoids detector-specific architectural modifications, and does not rely on target-domain data. Rather than emphasizing visual realism or random diversity, the proposed degradation strategy focuses on illumination factors that most directly affect detection robustness.

In summary, by constructing a task-aligned intermediate degraded domain, our method enables the model to learn representations that are robust to illumination variations.

## 3. Method

**Figure 1:**
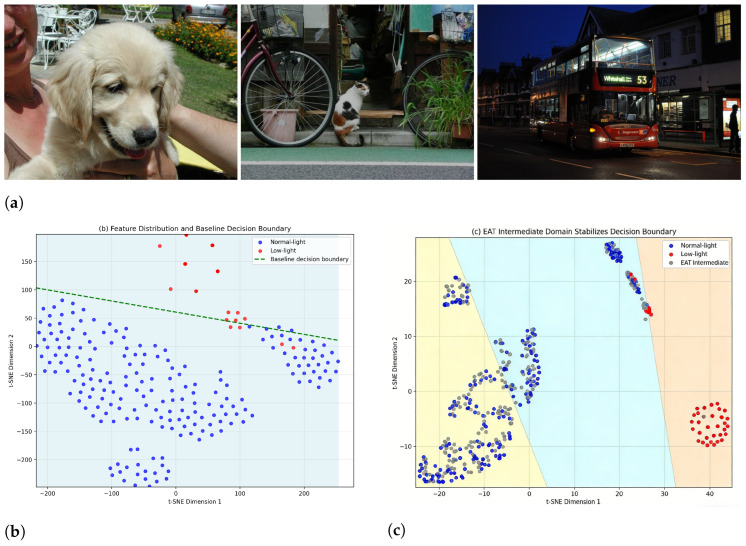
Schematic of the illumination gap and its mitigation via EAT. (**a**) Example normal-light, EAT-degraded, and real low-light images; (**b**) t-SNE visualization of baseline features: normal-light (blue) and low-light (red) features appear separated; (**c**) t-SNE visualization of EAT features: normal-light (blue) and intermediate-domain (gray) features are mixed, with low-light (red) features being in more compact clusters.

**Figure 2:**
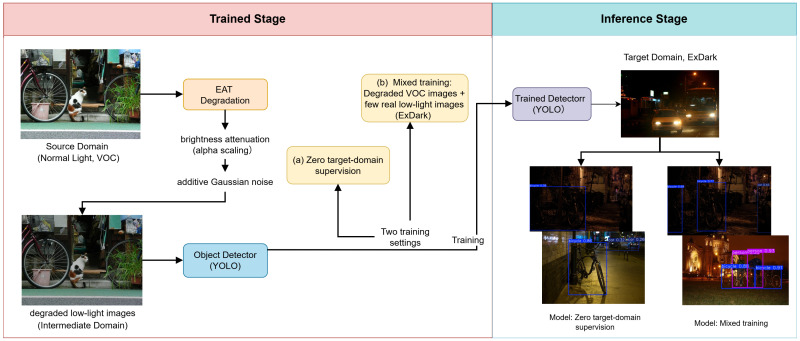
Overview of the proposed EAT framework. During training, normal-light images are degraded via brightness attenuation and additive noise. The detector is trained on these degraded images without target-domain data or architecture modifications. During inference, the trained detector directly processes real low-light images.

This section details the proposed task-oriented degradation-modeling method. We first elaborate on the core motivation—the illumination gap problem—and introduce a conceptual diagram (Figure 1). Subsequently, we formalize the degradation model, describe how attenuation parameters can be estimated from paired data via maximum likelihood estimation, and explain the training pipeline (Figure 2).

### 3.1. Motivation: The Illumination Gap Problem

Performance degradation in low-light object detection stems from the distribution shift between training and testing phases rather than visual quality degradation alone. Specifically, detection models are typically trained on normal-light images but deployed in low-light scenarios, where reduced global brightness and increased noise cause feature distribution shifts, making the original decision boundaries less effective.

We refer to this illumination-induced distribution mismatch as the illumination gap, as illustrated in Figure 1a. The essence of this gap lies in the fact that identical semantic content corresponds to different pixel statistics under varying lighting conditions, thereby affecting feature extraction and discrimination.

We have observed that real-world low-light degradation is not entirely random but follows certain patterns. For a large number of paired normal-light and low-light images, their relationship can be approximated as a combination of brightness attenuation and additive noise. Based on this observation, rather than directly fitting the real-world low-light distribution, we construct an intermediate degraded domain designed for detection training. In this domain, only the degradation factors critical to detection performance are retained. This design encourages the model to learn feature representations that are more robust to illumination variations. This is further validated by the feature distribution visualization in Figure 1b,c.

### 3.2. Conditional Degradation Model

#### 3.2.1. Degradation Model

During training, we apply the following degradation model to normal-light images:

(1) $$I^{\prime }\left(x,y\right)=\alpha I\left(x,y\right)+N\left(0,\sigma^{2}\right)$$

where I(x,y)∈[0,1] denotes the normalized normal-light image at spatial coordinate (x,y), α∈(0,1] is the illumination attenuation coefficient, $N(0,\sigma^{2})$ represents additive Gaussian noise, and $I^{\prime }\left(x,y\right)$ refers to the final degraded image. The corresponding object annotations are left unchanged to maintain consistent detection task constraints.

The degradation process mainly models two key low-light imaging factors, namely, global brightness variation and random noise perturbation, effectively capturing the dominant visual changes that degrade detection performance in real low-light scenes.

#### 3.2.2. Parameter Estimation

Instead of manually selecting degradation parameters, we estimate them from paired low-light data. For paired normal-light and low-light images $\left(I_{\mathrm{normal}},I_{\mathrm{low}}\right)$, we assume

(2) $$I_{\mathrm{low}}\left(x,y\right)=\alpha I_{\mathrm{normal}}\left(x,y\right)+\epsilon ,\;\epsilon ∼N\left(0,\sigma^{2}\right)$$

This model discards the intercept term since illumination degradation is mainly reflected in multiplicative brightness attenuation rather than a fixed additive offset. Under the zero-mean Gaussian noise hypothesis, maximum-likelihood estimation is equivalent to least squares regression, and the closed-form solution of the attenuation coefficient is derived as follows:

(3) $$\alpha =\frac{I_{\mathrm{normal}}^{⊤}\left(x,y\right)I_{\mathrm{low}}\left(x,y\right)}{I_{\mathrm{normal}}^{⊤}\left(x,y\right)I_{\mathrm{normal}}\left(x,y\right)}$$

Initial estimation. We performed initial estimation on 101 self-collected controlled paired images, obtaining α=0.7285±0.01.

Statistical estimation on a public dataset (LSD). To improve reproducibility, we performed validation using the public LSD dataset [19] (64,318 normal/low-light pairs). To reduce computational costs, we randomly sampled 5000 pairs (approximately 7.8% of the total data) using a fixed random seed (42). A robust filtering mechanism was then applied: we removed the darkest 10% of samples, retaining those with an α within the 5–95% percentile range and an $R^{2}\geq 0.9$. This yielded 115 valid pairs, with an estimated α=0.7450±0.1207, which is reasonably consistent with the initial estimate.

For the noise term, residual analysis shows a relatively small variance range. Following the sensor noise modeling approach of Unprocessing [20], we adopt additive Gaussian noise. To simplify training, we fix σ=0.01, which works stably in our experiments.

#### 3.2.3. Sensitivity Analysis

Detection performance exhibits a non-monotonic trend with respect to degradation intensity α: when degradation is too strong (α<0.6), image information loss is severe; when degradation is too weak (α>0.9), the illumination gap cannot be effectively reduced. Within the moderate degradation range, model performance remains relatively stable, indicating that the proposed degradation model is robust to parameter variations.

### 3.3. Task-Oriented Degradation Strategy

Unlike general data augmentation methods, EAT designs the degradation strategy from a task-oriented perspective, following these principles:

1. Do not pursue complete distribution fitting. In contrast to methods designed to approximate the real low-light distribution, we argue that detection performance is primarily influenced by key degradation factors rather than the full distribution. As a result, we construct a simplified intermediate degradation domain to reduce modeling complexity.
2. Focus on task-relevant degradation factors. Only brightness attenuation and additive noise—factors that significantly affect feature visibility—are retained, while secondary effects such as color shift and vignetting are not explicitly modeled, thereby reducing task-irrelevant interference.
3. Apply degradation only during training. The degradation process is introduced only in the training phase, with no additional processing during inference, thus incurring no computational overhead.

Relation to Photometric Augmentation: While EAT shares formal similarities with traditional photometric augmentation, they have different goals. Traditional methods apply random perturbations based on empirical heuristics (e.g., brightness or color changes), with the goal of increasing data diversity. In contrast, EAT estimates degradation parameters in a data-driven manner and constructs a degradation model with a well-defined structure, aiming to alleviate the illumination gap between training and testing.

### 3.4. Training Pipeline

We use YOLOv8 [21] as the baseline detector. The overall training and inference pipeline is illustrated in Figure 2. The process consists of the following steps:

1. Data preparation: Normal-light images and their annotations are used as input.
2. Degradation generation: The degradation transform $I^{\prime }\left(x,y\right)=\alpha I\left(x,y\right)+N\left(0,\sigma^{2}\right)$ is applied to training images.
3. Model training: The model is trained using degraded images and original annotations, optimizing the standard detection loss.
4. Inference: The original images are directly fed into the model for prediction, without any degradation or enhancement operations.

All training hyperparameters are kept consistent with the baseline: the batch size is 16, the input dimensions are 640 × 640, the optimizer used is AdamW, and the initial learning rate is 0.001, and the cosine annealing scheduler is used. Data preprocessing and degradation generation use a fixed random seed (42), while detector training follows the default YOLO settings. This ensures that performance differences are solely attributable to the degradation strategy.

The degradation process is only introduced during training, and it is completely removed during inference.

#### Computational Overhead

The degradation operation involves only element-wise multiplication, addition, and Gaussian noise generation, applied online during training. In our experiments, compared to the baseline training (100 epochs on TLD), the extra time per epoch is less than 1% of the total training time. More importantly, the degradation module is completely removed at the inference stage, so EAT introduces **zero additional computational cost** during deployment.

## 4. Experiments

This section presents a systematic evaluation of the proposed Exposure-Aware Training (EAT) method. The experiments were conducted from two perspectives, namely, in-domain performance and cross-domain generalization, with ablation studies and comparative experiments further validating the effectiveness and robustness of the method.

### 4.1. Experimental Setup

TLD Traffic Dataset (In-Domain Evaluation): This dataset contains 4941 normal-light traffic images, covering seven categories: blank, countdown_blank, countdown_go, countdown_stop, crossing, go, and stop. The data is split 8:2 into training (3941 images) and validation (1000 images). To analyze performance under different lighting conditions, we selected the darkest 30% of all images according to the grayscale mean and randomly sampled 500 images as the dark validation subset.

VOC2012 Dataset (Cross-Domain Training): This dataset was used as the normal-light training source for cross-domain experiments. We retained the 10 categories overlapping with ExDark: bicycle, boat, bottle, bus, car, cat, chair, dog, motorbike, and person. After an 8:2 split, the dataset contained 6892 training images and 1723 validation images.

ExDark Dataset (Cross-Domain Testing and Data-Scarce Evaluation): ExDark contains 7363 real low-light images. After filtering, the data was split into training (5066), validation (633), and test (635) sets in an 8:1:1 ratio. This dataset was used for (1) cross-domain testing (no ExDark data were used during training) and (2) data-scarce scenarios (mixed training with EAT synthetic data).

LSD Public Dataset (Parameter Estimation): This dataset is used to estimate degradation model parameters [19].

Evaluation Metrics and Training Configuration: Standard object detection metrics were adopted in the experiments, including mAP@0.5 and mAP@0.5:0.95. All models were implemented based on YOLOv8n with an input resolution of 640 × 640 and a batch size 16 and trained for 100 epochs using the AdamW optimizer with an initial learning rate of 0.001, and the cosine annealing strategy was used. In all the experiments, a fixed random seed of 42 was used, and default YOLO settings were followed to ensure reproducibility.

### 4.2. Comparison with Existing Low-Light Detection and Augmentation Strategies

To better position EAT within the low-light detection literature, we compare it with representative low-light detection methods and modern augmentation strategies. Rather than focusing solely on absolute $mAP_{50}$ values, we additionally consider architectural complexity, target-domain dependency, and inference-stage overhead.

#### 4.2.1. Comparison with Existing Low-Light Detection Methods

Existing low-light object detection methods mainly fall into three categories: image enhancement, detector redesign, and unsupervised domain adaptation.

Enhancement-based methods improve image visibility before detection and often achieve strong performance under fully supervised settings. For example, FE-YOLO [22] combines a Fourier enhancement network with YOLOv3 and yields 68.9% $mAP_{50}$ on ExDark. However, these approaches usually require additional enhancement modules during inference, increasing computational costs.

Detector redesign methods incorporate low-light adaptation directly into the detector architecture. Recent approaches such as FE-YOLOX [23], LMD-YOLO [24], RFSC-Net [25], and SCL-YOLOv11 [26] achieved an $mAP_{50}$ of around 67–69% on ExDark through feature enhancement, multi-scale fusion, or customized detection heads. Although effective, these methods rely on detector-specific modifications and increased architectural complexity.

Domain adaptation methods reduce the distribution gap between normal-light and low-light data through feature alignment. LIDA-YOLO [27], for instance, uses unlabeled target-domain images and can achieve a 56.65% $mAP_{50}$ on ExDark. Nevertheless, target-domain data are still required during training.

Different from the methods above, EAT adopts a lightweight training-stage degradation strategy without modifying the detector architecture or introducing additional inference costs. As shown in Table 2, EAT achieved a 68.6% $mAP_{50}$ on ExDark, which is comparable to several recent low-light detectors, while maintaining a simpler training and deployment pipeline. These results suggest that competitive low-light robustness can still be achieved without detector redesign or test-time enhancement.

#### 4.2.2. Difference from Modern Augmentation Methods

EAT also differs fundamentally from modern general-purpose augmentation methods such as AugMix [16] and RandAugment [28]. Table 3 summarizes the key paradigm-level differences between these approaches.

The primary goal of AugMix and RandAugment is to improve general robustness against unknown perturbations by increasing data diversity through randomly combined augmentation strategies. In contrast, EAT specifically targets the distribution shift between normal-light and low-light conditions.

Therefore, EAT is not intended to be a general data augmentation method; rather, it is a degradation modeling strategy designed for low-light object detection. Its core objective is not to increase randomness but to reduce the illumination distribution gap between training and testing environments.

### 4.3. Ablation Study on Degradation Modeling

To systematically verify the effectiveness of the proposed brightness attenuation (α) and Gaussian noise approaches in low-light object detection, we conducted ablation studies on both the TLD and VOC datasets. We first examined single-factor and dual-factor degradation combinations (Section 4.3.1) and then investigated the effect of mixing degraded data directly into the training set (Section 4.3.2).

#### 4.3.1. Effect of Single and Combined Degradation

To evaluate the influence of illumination attenuation (Alpha) and Gaussian noise (Gauss) on low-light object detection, ablation experiments were conducted using the TLD and VOC datasets. The performances of single-factor degradation, dual-factor degradation, and the baseline model are compared in Table 4.

The single-factor results show that both Alpha and Gauss can improve detection performance to different extents. Among them, Alpha achieved the highest $mAP_{50}$, 55.9, on the TLD dataset, improving the baseline by 0.6. Gauss achieved the best performance on the VOC dataset, with an $mAP_{50}$ of 64.1. In contrast, Gamma only provided a marginal improvement, while Poisson noise significantly reduced detection performance, especially on the TLD dataset.

For dual-factor degradations, Alpha&Gauss achieved relatively stable performance across both datasets. The combination reached an $mAP_{50}$ of 56.7 on the TLD dataset while maintaining comparable performance on VOC without obvious degradation. By comparison, Gauss&Poisson also achieved relatively high performance on TLD but suffered a substantial decline after transferring to the VOC dataset, indicating weaker stability.

Overall, moderate illumination attenuation combined with Gaussian noise provides more stable improvements for low-light detection, whereas excessively strong or unstable noise perturbations may damage object structure information and negatively affect detection accuracy.

#### 4.3.2. Effect of Mixing Degraded Data in Training

Table 5 presents the results of mixed training by incorporating degraded images into the original training set.

Overall, most degradation strategies improve detection performance relative to the baseline model, although the effectiveness varies across datasets and degradation types. The improvements on the VOC dataset are generally more noticeable than those on TLD. One possible reason is that VOC mainly contains normal-light images, whereas TLD already includes mixed illumination conditions. Excessive degradation may therefore reduce the consistency of certain samples in TLD.

For single-factor degradation schemes, all four strategies achieved stable performance gains on the VOC dataset. Among them, +Gauss obtained the highest $mAP_{50}$, 67.5, on VOC, while both +Gamma and +Poisson reached the peak $mAP_{50}$, 56.9, on the TLD dataset.

In terms of dual-factor combinations, +Alpha&Gauss and +Gauss&Poisson both achieved the highest $mAP_{50}$, 56.9, on TLD. Nevertheless, +Alpha&Gauss exhibited better cross-dataset stability and maintained a competitive $mAP_{50}$ of 66.4 on VOC. In comparison, +Gauss&Poisson suffered a noticeable performance drop when transferred to the VOC dataset. Other combinations, including +Alpha&Gamma and +Alpha&Poisson, can boost performance on VOC but cause accuracy degradation on TLD.

Compared with models trained merely on fully degraded datasets, mixed training yields more steady performance improvements. Furthermore, dual-factor degradation methods deliver more prominent gains on the TLD dataset, while single-factor strategies achieve slightly better overall results on the VOC dataset.

**Figure 3:**
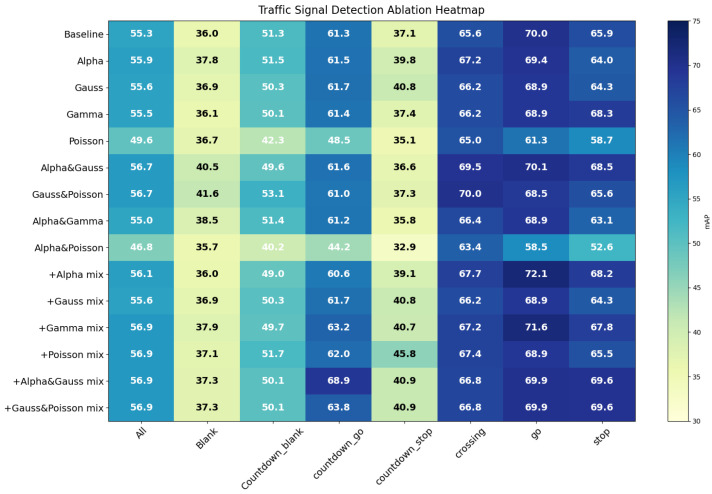
Category-level performance heatmap of different strategies on TLD dataset.

**Figure 4:**
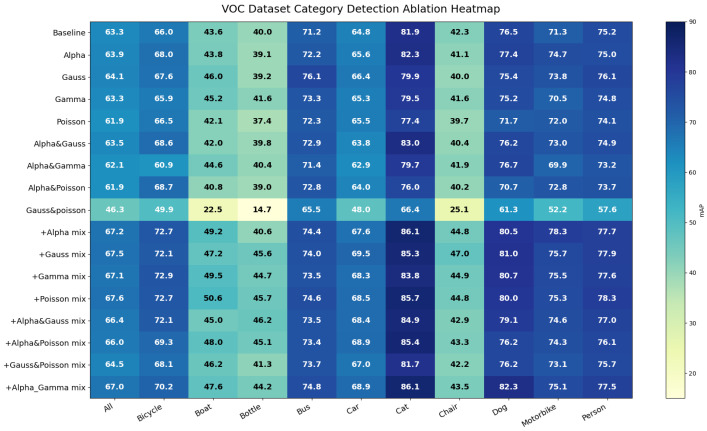
Category-level performance heatmap of different strategies on VOC dataset.

Figure 3 and Figure 4 further present the category-level heatmap results under different degradation strategies. On the TLD dataset, Alpha&Gauss and Gauss&Poisson achieved the strongest overall category-level performance. Compared with Gauss&Poisson, Alpha&Gauss provides more balanced improvements across background, crossing, and traffic-signal-related categories. Mixed-training strategies do not consistently outperform direct dual-factor degradations, suggesting that carefully designed degradation combinations may already provide sufficient low-light adaptation.

Different degradation strategies also exhibit distinct category-level behaviors. Categories such as Blank, Crossing, and the countdown series show relatively high sensitivity to illumination degradation. Most strategies improve the Blank and Crossing categories, while Alpha&Gauss produces comparatively stable gains across these classes. For countdown_go and countdown_stop, degradation strategies involving Poisson noise lead to noticeable performance drops, indicating that strong stochastic noise may interfere with low-contrast small-object features.

On the VOC dataset, single-factor degradation approaches provide relatively limited gains, while the direct combination of Gaussian and Poisson noise causes substantial performance degradation. By contrast, mixed-training strategies improve overall robustness more consistently, with more noticeable gains observed for challenging categories such as Boat, Bottle, and Chair.

Overall, the heatmap results indicate that the effectiveness of degradation strategies depends not only on degradation composition but also on category characteristics and dataset illumination distribution.

### 4.4. Cross-Dataset Evaluation

To further evaluate the cross-dataset adaptability of the proposed degradation strategy, additional experiments were conducted on the ExDark dataset using degradation configurations derived from the VOC and TLD datasets.The results are presented in Table 6.

The baseline model achieved an overall $mAP_{50}$ of 47.1 on ExDark. After we introduced single-factor degradation strategies generated from the VOC dataset, including +VOC_Alpha, +VOC_Gauss, and +VOC_Poisson, the overall $mAP_{50}$ increased to above 54, corresponding to improvements of more than 7 percentage points over the baseline. These results indicate that moderate degradation generated from normal-light datasets can effectively improve low-light detection performance.

Dual-factor degradation strategies further improve detection accuracy. Among them, +VOC_Gauss&Poisson achieved an overall $mAP_{50}$ of 59.1, improving the baseline by approximately 12 percentage points. Compared with single-factor degradation approaches, the dual-factor combinations generally provide stronger improvements on ExDark, suggesting that compound degradation patterns may better simulate illumination variation in low-light environments.

We further evaluate degradation strategies derived from the TLD dataset. Compared with VOC-based strategies, TLD-based degradation approaches achieved better cross-dataset performance on ExDark. In particular, +TLD+TLD_Alpha&Gauss achieved the highest overall $mAP_{50}$ of 60.3, while +TLD_Alpha&Gauss reached 59.5. Since TLD already contains mixed illumination conditions, the estimated degradation statistics may be more consistent with the illumination distribution of ExDark.

Overall, the results demonstrate that the proposed degradation strategy can maintain stable effectiveness across different datasets and illumination distributions. Moderate degradation combinations, especially those involving illumination attenuation and Gaussian noise, provide more consistent improvements under cross-dataset, low-light evaluation conditions.

### 4.5. Supplementary Validation and Robustness Analysis

Finally, the proposed strategy was further validated on YOLOv8 and Faster R-CNN detectors. As shown in Table 7, EAT effectively improves low-light object detection performance for both mainstream detectors. Meanwhile, the proposed method introduces no additional computation overhead during the inference stage, demonstrating its wide applicability and efficiency.

## 5. Discussion

### 5.1. Rationality and Limitations of Degradation Modeling

In this work, we adopted a linear degradation model, $I^{\prime }\left(x,y\right)=\alpha I\left(x,y\right)+\epsilon$, to describe the low-light imaging process. The experimental results show that the model achieved a good fit on paired data ($R^{2}>0.9$), indicating that global illumination attenuation can be effectively modeled from a statistical perspective. However, real low-light environments are usually more complex and may involve nonlinear responses, spatially non-uniform illumination, and sensor-related noise. As a result, the proposed linear model should be regarded as a simplified approximation whose primary purpose is to capture the dominant factors affecting detection performance rather than to precisely reproduce the real imaging process.

Notably, even without explicitly modeling nonlinear degradation, EAT maintains relatively stable performance under different illumination conditions. As shown in Table A2, when γ increases from 1.0 to 2.5, the $mAP_{50}$ of EAT decreases, while the Baseline also decreases, with EAT consistently remaining above the Baseline. This suggests that the method does not rely on accurate physical modeling but instead improves robustness by constructing an intermediate degraded domain that encourages illumination-invariant feature learning.

### 5.2. Parameter Estimation and Method Robustness

The illumination attenuation parameter α is estimated from a small amount of paired data, providing a certain degree of physical interpretability for degradation strength. It should be emphasized that paired data are not required during training; they are only used to provide a reasonable initialization range for the parameter. The estimated α distribution on the public dataset remains relatively consistent, with a mean value of around 0.74.

Our sensitivity analysis (Table A3) further shows that when α varies within the range [0.5, 0.8], the $mAP_{50}$ of EAT remains stable between 0.590 and 0.592, whereas the Baseline decreases from 0.590 to 0.580. The results imply that EAT is more robust to variations in degradation strength. As a result, even in practical scenarios where paired data are unavailable, the parameter can still be approximated using empirical ranges or simple statistical information. This property improves the practicality of the method in real-world applications.

### 5.3. Computational Efficiency

The proposed EAT method introduces negligible training overhead because the degradation operation ($I^{\prime }=\alpha I+N\left(0,\sigma^{2}\right)$) is applied online and involves only element-wise arithmetic. In our experiments, the extra time per epoch compared to baseline training is less than 1% of the total training time. More importantly, the degradation module is completely removed at inference, resulting in **zero additional computational cost** during deployment. This makes EAT particularly suitable for resource-constrained applications where low inference latency is critical.

### 5.4. Positioning of the Method and Relation to Existing Approaches

Existing low-light detection strategies, including enhancement-based methods, architectural modification methods, and domain adaptation methods, improve robustness from different perspectives, but they often rely on target-domain data, introduce additional architectural complexity, or increase inference cost. EAT addresses the problem from a degradation-based training perspective: the detector is exposed to controlled illumination degradation during training, with degradation parameters estimated from paired data.

As shown in Table 7, EAT produces consistent improvements on both YOLOv8 and Faster R-CNN, with relative gains of approximately 3.4–3.5%, indicating that the strategy is not tied to a specific detector architecture. The comparison in Table 2 is not intended to suggest that EAT outperforms enhancement-based or domain adaptation methods in absolute performance, since those methods are developed under different supervision settings, such as fully supervised training or access to unlabeled target-domain data. Instead, EAT focuses on a more constrained setting in which no target-domain images are used during training, the detector architecture remains unchanged, and no additional inference-stage computation is introduced. In this setting, EAT serves as a complementary training strategy for improving robustness under illumination variation.

### 5.5. Limitations and Future Work

Several limitations still remain in the current study. The proposed linear degradation model cannot adequately describe spatially non-uniform illumination, such as local shadows or highlights. In addition, excessively strong compound degradation may substantially damage detection performance. As shown in Table 4, the Gauss_poisson strategy reduces the overall VOC $mAP_{50}$ from 63.3% to 46.3%, with particularly severe degradation on small-object categories, such as “Boat” (43.6% → 22.5%) and “Bottle” (40.0% → 14.7%). This observation suggests a trade-off between degradation strength and information preservation. In scenes containing very small objects or heavy occlusion, the degradation process may further obscure already-limited visual features.

We also observed failure cases under highly challenging conditions, including extremely small objects (e.g., distant pedestrians under low-light conditions) and scenes with highly uneven illumination, such as strong local shadows adjacent to bright regions. In these situations, global linear degradation may excessively darken already low-visibility regions while insufficiently affecting brighter areas.

Future work may explore spatially adaptive degradation, nonlinear imaging models, adaptive selection of degradation strength, and extension of the strategy to other vision tasks such as segmentation and tracking.

## 6. Conclusions

This paper presents Exposure-Aware Training (EAT), a lightweight degradation-based strategy for improving low-light object detection. By introducing illumination attenuation and additive noise during training, EAT improves robustness to illumination variation without modifying detector architectures or introducing additional inference costs.

Experiments conducted on multiple datasets show that the proposed strategy consistently improves performance in both cross-domain and mixed-training settings. The improvements are particularly noticeable for illumination-sensitive object categories and remain stable across different detectors.

Although the current degradation model is intentionally simple and does not fully describe complex real-world low-light imaging effects, the results suggest that task-oriented degradation modeling can still provide meaningful robustness gains for low-light detection. Future work will explore more realistic degradation processes and adaptive illumination-modeling strategies.

## Figures and Tables

**Table 1:** Comparison of different method categories.

Method Category	Test-Time Overhead	Requires Target-Domain Annotations	Design Basis
Image Enhancement	Yes	No	Visual quality
Architecture Modification	Yes	No	Architecture design
General Augmentation	No	No	Data diversity
Domain Adaptation	No	Yes	Distribution alignment
EAT (Ours)	No	No	Task-oriented degradation

**Table 2:** Performance comparison with existing low-light detection methods on the ExDark dataset.

Method	${\mathrm{mAP}}_{50}$	Arch. Change	Inf. Overhead
FE-YOLOX [23]	69.1%	Yes	Yes
LMD-YOLO [24]	69.1%	Yes	Yes
FE-YOLO [22]	68.9%	No	Yes
3L-YOLO [9]	68.8%	Yes	Yes
EAT (Ours)	68.6%	No	No
RFSC-Net [25]	∼67.9%	Yes	No
SCL-YOLOv11 [26]	67.6%	Yes	Yes
Domain adaptation [27]	56.65%	Yes	Yes

Note: Arch. change denotes whether the detector architecture is modified; Inf. overhead denotes whether additional computational costs exist during the inference phase. Estimated value of RFSC-Net is calculated according to the reported 2.0% performance gain over YOLOv8n baseline, and the result is approximate.

**Table 3:** Paradigm-levelcomparison between EAT and modern augmentation methods.

Aspect	AugMix/RandAugment	EAT (Ours)
Primary objective	General robustness	Low-light generalization
Core mechanism	Random augmentation combinations	Illumination degradation modeling
Parameter source	Random/heuristic	Data-driven estimation
Domain-specific design	No	Yes

**Table 4:** Comparison of single-factor and dual-factor degradation methods.

Method	Degradation Formula	TLD Dataset					VOC Dataset			
		${\mathrm{mAP}}_{50}$	${\mathrm{mAP}}_{50–90}$	P	R		${\mathrm{mAP}}_{50}$	${\mathrm{mAP}}_{50–90}$	P	R
Baseline	$I^{\prime }\left(x,y\right)=I\left(x,y\right)$	55.3	40.4	46.6	87.3		63.3	44.7	71.3	55.8
Alpha	$I^{\prime }\left(x,y\right)=\alpha I\left(x,y\right)$	55.9	41.5	48.2	80.9		63.9	44.8	70.1	57.7
Gauss	$I^{\prime }\left(x,y\right)=I\left(x,y\right)+N\left(0,\sigma^{2}\right)$	55.6	40.8	47.3	83.3		64.1	45.1	69.6	57.6
Gamma	$I^{\prime }\left(x,y\right)=I{\left(x,y\right)}^{\gamma }$	55.5	40.7	46.5	86.8		63.3	44.0	73.9	54.8
Poisson	$I^{\prime }\left(x,y\right)=I\left(x,y\right)+N_{\mathrm{Poisson}}$	49.6	33.1	37.2	74.4		61.9	42.9	72.8	53.9
Alpha + Gauss	$I^{\prime }\left(x,y\right)=\alpha I\left(x,y\right)+N\left(0,\sigma^{2}\right)$	56.7	41.5	48.2	82.7		63.5	44.1	72.4	56.1
Gauss + Poisson	$I^{\prime }\left(x,y\right)=I\left(x,y\right)+N\left(0,\sigma^{2}\right)+N_{\mathrm{Poisson}}$	56.7	41.0	47.6	83.0		46.3	29.3	57.8	40.9
Alpha + Gamma	$I^{\prime }\left(x,y\right)=\alpha I{\left(x,y\right)}^{\gamma }$	55.0	39.9	45.7	83.4		62.1	43.3	71.2	55.0
Alpha + Poisson	$I^{\prime }\left(x,y\right)=\alpha I\left(x,y\right)+N_{\mathrm{Poisson}}$	46.8	30.9	32.7	63.8		61.9	43.4	69.8	55.4

**Table 5:** Overall detection performance of different individual and paired degradation strategies.

Method	TLD Dataset					VOC Dataset			
	${\mathrm{mAP}}_{50}$	${\mathrm{mAP}}_{50–90}$	P	R		${\mathrm{mAP}}_{50}$	${\mathrm{mAP}}_{50–90}$	P	R
Baseline	55.3	40.4	46.6	87.3		63.3	44.7	71.3	55.8
+Alpha	56.1	42.9	48.0	81.0		67.2	48.3	75.5	59.2
+Gauss	55.6	40.8	47.3	83.3		67.5	47.7	75.1	60.6
+Gamma	56.9	43.4	48.0	82.4		67.2	47.7	77.3	58.3
+Poisson	56.9	41.9	47.0	78.9		67.6	48.4	74.4	60.5
+Alpha&Gauss	56.9	43.5	49.3	81.9		66.4	47.4	75.4	58.9
+Gauss&Poisson	56.9	43.5	49.3	81.9		64.5	45.5	70.4	58.1
+Alpha&Gamma	55.0	39.9	45.7	83.4		67.0	47.7	76.4	59.3
+Alpha&Poisson	46.8	30.9	32.7	63.8		66.0	47.0	74.5	59.3

**Table 6:** Cross-domain testing results on ExDark dataset.

Method	All	Bicycle	Boat	Bottle	Bus	Car	Cat	Chair	Dog	Motorbike	Person
Baseline	47.1	52.5	57.3	46.5	51.5	51.6	49.4	36.6	46.9	37.3	41.4
+VOC_Alpha	54.6	63.5	66.6	52.2	59.1	54.0	57.7	44.8	57.6	49.6	40.3
+VOC_Gauss	54.4	61.3	63.2	52.0	60.0	55.8	59.1	43.9	58.2	49.8	40.5
+VOC_Gamma	49.6	57.0	58.7	45.4	56.1	58.1	49.7	38.4	48.0	45.4	38.7
+VOC_Poisson	54.3	59.6	65.6	49.8	60.3	55.7	57.6	45.0	59.8	49.1	40.4
+VOC_Alpha&Gauss	55.0	67.9	64.4	47.2	57.9	58.8	59.9	47.1	57.8	49.6	39.4
+VOC_Alpha&Gamma	54.5	61.0	64.4	52.1	59.6	58.1	61.9	41.8	59.1	50.1	37.1
+VOC_Alpha&Poisson	55.6	63.2	64.5	52.7	61.8	55.2	60.4	46.4	57.6	48.0	46.6
+VOC_Gauss&Poisson	59.1	67.7	68.6	57.3	62.4	55.2	58.6	53.4	64.3	55.8	47.7
+TLD_Alpha&Gauss	59.5	69.4	71.0	55.5	65.5	57.2	61.7	49.4	65.4	54.0	45.4
+TLD+TLD_Alpha&Gauss	60.3	67.3	67.1	57.7	67.0	64.2	59.9	52.5	59.1	58.9	49.2

**Table 7:** Mixed training results in the data-scarce scenario for two detectors.

Detector	Model	Normal Test ${\mathrm{mAP}}_{50}$	Low-Light Test ${\mathrm{mAP}}_{50}$
YOLOv8	Baseline	0.410	0.594
	EAT-Augmented	0.707	0.614
Faster R-CNN	Baseline	0.417	0.490
	EAT-Augmented	0.727	0.507

Notes: For YOLOv8, EAT-Augmented improves low-light test $mAP_{50}$ by +0.020 (+3.4%), while for Faster R-CNN, the improvement is +0.017 (+3.5%). Both detectors also show large gains on the normal-light test set. Relative improvement is computed as $\Delta \left(\%\right)=\left({\mathrm{mAP}}_{\mathrm{EAT}}-{\mathrm{mAP}}_{\mathrm{baseline}}\right)/{\mathrm{mAP}}_{\mathrm{baseline}}\times 100\%$.

## Data Availability

The data that support the findings of this study are available from the corresponding author upon reasonable request. The VOC2012 dataset [29] is publicly available at http://host.robots.ox.ac.uk/pascal/VOC/ (accessed on 5 April 2026). The ExDark dataset [3] is publicly available at https://github.com/cs-chan/Exclusively-Dark-Image-Dataset (accessed on 5 March 2026). The LSD dataset [19] is publicly available at https://github.com/sharif-apu/LSD-TFFormer (accessed on 19 May 2026).

## Abbreviations

The following abbreviations are used in this manuscript:

EAT	Exposure-Aware Training
mAP	Mean Average Precision
MLE	Maximum-Likelihood Estimation
TLD	Traffic-Light Dataset (a proprietary paired normal/low-light traffic dataset)
LSD	Low-Light Smartphone Dataset
VOC	Visual Object Classes
SOTA	State of the Art

## Appendix A. Sensitivity to Random Seeds

**Table A1:** Performance comparison under different random seeds.

Random Seed	Baseline ${\mathrm{mAP}}_{50}$	EAT ${\mathrm{mAP}}_{50}$
0	0.607	0.610
42	0.590	0.601
123	0.595	0.603
234	0.601	0.606
345	0.602	0.606
456	0.595	0.603
567	0.602	0.608

## Appendix B. Generalization to Nonlinear Illumination Changes

**Table A2:** Detection performance under different gamma values.

γ	Baseline ${\mathrm{mAP}}_{50}$	EAT ${\mathrm{mAP}}_{50}$
0.8	0.617	0.521
1.0	0.607	0.510
1.5	0.531	0.542
2.0	0.448	0.459
2.5	0.405	0.414

## Appendix C. Sensitivity to Degradation Intensity α

**Table A3:** Performance sensitivity to degradation intensity α on the TLD dark subset.

α	Baseline ${\mathrm{mAP}}_{50}$	EAT ${\mathrm{mAP}}_{50}$
0.5	0.590	0.592
0.6	0.586	0.591
0.7	0.583	0.590
0.8	0.580	0.590
